# The Effect of Hydrogen Sulfide and Reactive Sulfur Species on Bacterial Virulence and Antibiotic Sensitivity

**DOI:** 10.3390/ijms27156983

**Published:** 2026-08-03

**Authors:** Galina Smirnova, Aleksey Tyulenev, Lyubov Sutormina, Elena Forte, Vitaliy B. Borisov, Oleg Oktyabrsky

**Affiliations:** 1 Institute of Ecology and Genetics of Microorganisms, Perm Federal Research Center of the Ural Branch of the Russian Academy of Sciences, 614081 Perm, Russia; smirnova@iegm.ru (G.S.); leksey333@yandex.ru (A.T.); lyubov-sutormina@mail.ru (L.S.); oktyabr@iegm.ru (O.O.); 2 Department of Biochemical Sciences, Sapienza University of Rome, 00185 Rome, Italy; elena.forte@uniroma1.it; 3 Belozersky Institute of Physico-Chemical Biology, Lomonosov Moscow State University, Leninskie Gory, 119991 Moscow, Russia; 4 Faculty of Bioengineering and Bioinformatics, Lomonosov Moscow State University, Leninskie Gory, 119991 Moscow, Russia

**Keywords:** bacteria, endogenous and exogenous H_2_S, reactive sulfur species, H_2_S/RSS homeostasis, virulence, antibiotic sensitivity

## Abstract

Recent research has demonstrated the important role of hydrogen sulfide (H_2_S) and its derivatives, reactive sulfur species (RSS), as modulators of various redox-regulated physiological processes in bacteria. Bacterial cells are equipped with enzymes that synthesize and catabolize H_2_S and RSS, and sensors that control the expression of genes whose products ensure the maintenance of safe levels of these compounds in cells and the survival of bacteria in the host environment. With the rapid growth of resistant pathogens, the impact of H_2_S and RSS on bacterial virulence and antibiotic sensitivity is attracting increasing attention. The possibility of enhancing the efficacy of widely used antibiotics by artificially modulating H_2_S levels is being explored. This review summarizes current data on the sources and conditions of endogenous H_2_S and RSS production, the molecular mechanisms of action of various concentrations of exogenous and endogenous H_2_S, and the regulatory factors that control the expression of virulence and antibiotic resistance genes. Possible reasons for the conflicting results obtained by different research groups regarding the possibility of modulating bacterial sensitivity to antibiotics by altering the production of endogenous H_2_S are discussed.

## 1. Introduction

In mammals, H_2_S, like NO and CO, is a member of the class of gasotransmitters, endogenously produced gaseous molecules with well-defined physiological regulatory functions [1]. These molecules are endogenously generated by specific enzymes and freely diffuse across cell membranes, playing a key role in numerous signaling pathways and modulating physiological functions [2,3]. Gasotransmitters can modulate apoptosis and angiogenesis, the immune response and cellular redox status, reduce oxidative stress and inhibit pathogen growth [4]. The regulatory properties of gasotransmitters are used in the development of new approaches for the treatment of cardiovascular and neurological diseases, inflammatory effects, as well as for tissue regeneration and cancer therapy [4].

Many bacteria are also capable of producing these small molecules, can respond to their presence in the host organism, and use them as signals to regulate physiological functions [5]. In recent years, interest in the effects of exogenous and endogenous H_2_S on physiological processes in bacteria has increased significantly, especially after the discovery of its ability to modulate bacterial sensitivity to antibiotics and oxidative stress [6,7]. The presence in bacteria of genes encoding enzymes involved in the synthesis, catabolism, and sensing of H_2_S and reactive sulfur species (RSS), as well as H_2_S- and RSS-dependent signaling pathways and transcription factors controlling virulence and antibiotic resistance genes [8], suggests that fine-tuning of H_2_S and RSS levels may play an important role in pathophysiology. Developing ways to influence bacterial virulence and antibiotic sensitivity by targeted modulation of H_2_S and RSS levels is a very attractive idea for enhancing the efficacy of existing antibiotics, which has stimulated intensified research in this area. A significant amount of data has been accumulated, but numerous issues remain poorly understood, including the specific mechanisms of action and regulatory functions of H_2_S and its derivatives in different bacterial species, the role of bacterial physiological state, H_2_S sources, and antibiotic types, as well as the possibility of creating universal adjuvants. Some of the data obtained is contradictory, even for bacteria of the same species. In this review, we summarize recent research and examine issues related to H_2_S and RSS production in bacteria, their role in bacterial physiology, and their impact on bacterial virulence and antibiotic sensitivity. Based on an analysis of the available literature, we also attempt to elucidate the causes of the existing contradictory findings and identify ways to resolve them.

## 2. The Effect of H_2_S on Cells Depends on Concentration

Although the term “hydrogen sulfide” is most often used in the literature, in aqueous solutions H_2_S is a weak acid that dissociates into the HS^−^ and S^2−^ anions. Under physiological conditions (pH 7.4), “H_2_S” exists predominantly as the HS^−^ anion (∼69–73%), H_2_S (∼27–31%), and a small amount of S^2−^, which are collectively referred to as the “sulfide pool” [9,10]. The cumulative biological effect of the sulfide pool is usually considered. While concentrations of hydrogen sulfide ranging from nanomolar to micromolar can play a cytoprotective role, higher levels are generally toxic to cells [5,10]. Exogenous H_2_S (80 μM) disrupted redox and energy homeostasis in *Acinetobacter baumannii*, stimulated membrane depolarization, decreased ATP levels, and inhibited the expression of superoxide dismutase and enzymes related to glutathione metabolism [11]. In *Shewanella oneidensis*, catalase KatB was shown to be inhibited in the presence of H_2_S [12]. Consistently, H_2_S released by sodium hydrosulfide (NaHS) inhibited catalase and superoxide dismutase, disrupted the redox balance and suppressed the growth of *E. coli* in a dose-dependent manner [13].

**Figure 1:**
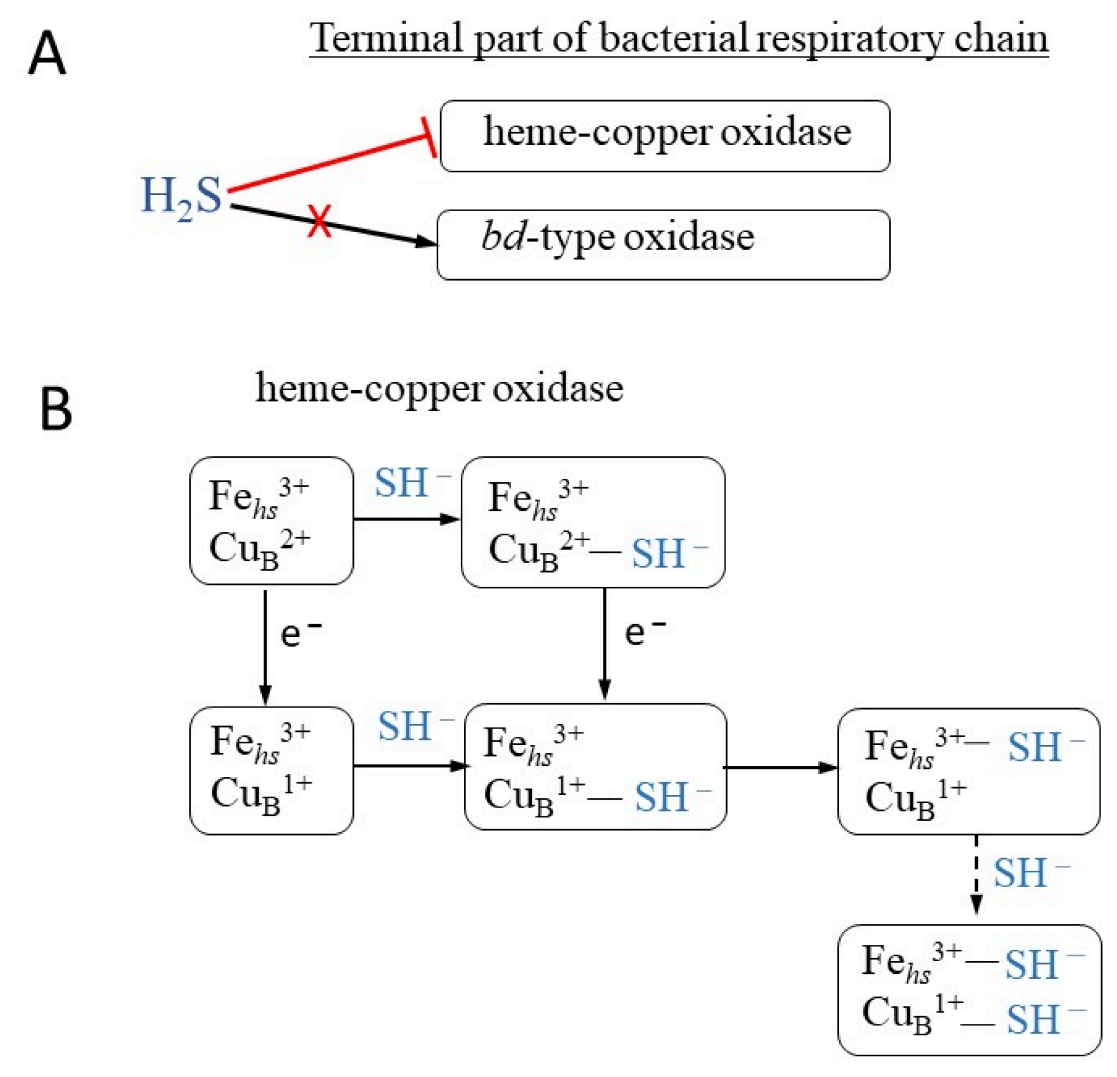
H_2_S and bacterial terminal oxidases. (**A**) Graphic sketch showing that H_2_S inhibits a heme-copper oxidase but does not affect the function of a *bd*-type oxidase. The red ‘X’ symbol indicates that H_2_S has no effect on a *bd*-type oxidase. (**B**) Proposed molecular mechanism for H_2_S-mediated inhibition of a heme-copper oxidase. Shown are different states of the binuclear center, which comprises a high-spin heme (Fe*_hs_*) and a copper ion (Cu_B_).

The respiratory chain of *E. coli* contains three terminal oxidases: cytochromes *bo*_3_, *bd*-I, and *bd*-II. Cytochrome *bo*_3_ belongs to the heme-copper oxidase superfamily, whereas copper-lacking cytochromes *bd*-I and *bd*-II are members of the *bd*-type oxidase superfamily [14,15]. Low micromolar concentrations of sulfide (half-maximal inhibitory concentration IC_50_~1–3 μM H_2_S) were reported to exhibit a toxic effect by inhibiting cytochromes *bo*_3_ but stimulate the transition of respiration to cytochromes *bd*-I and *bd*-II [16,17]. The transition to the less energetically efficient but sulfide-insensitive *bd*-type terminal oxidase allows *E. coli* to grow in the presence of H_2_S. However, at concentrations above 0.1 mM, exogenous Na_2_S decreased the specific growth rate of *E. coli* [18]. In agreement with [18], in the presence of sulfide-producing *Desulfovibrio piger*, PhsABC- and AsrABC-mediated H_2_S production by *Salmonella enterica* serovar Typhimurium was linked to inhibition of cytochrome *bd*-mediated respiration of *E. coli* in the murine large intestine [19]. Consistent with the *E. coli* data described above, in the presence of high levels of H_2_S, the activity of the cyanide-insensitive *bd*-type oxidase in *Pseudomonas aeruginosa* was unaltered and expression of this enzyme was enhanced. In contrast, the overall O_2_ consumption of all heme-copper terminal oxidases in *P. aeruginosa* was severely impaired by sulfide [20]. In *Shewanella oneidensis*, inhibition of the *cbb*_3_-type heme-copper oxidase by 0.25 mM H_2_S was also observed [12]. It appears that when both a heme-copper oxidase and a *bd*-type oxidase are present in the terminal part of the respiratory chain, bacteria respond to sulfide stress by upregulating cytochrome *bd*, as H_2_S inhibits the heme-copper oxidase (Figure 1A). In the absence of H_2_S and other stressors, bacterial aerobic respiration is primarily mediated by the heme-copper oxidase, which provides a higher energetic yield.

In general, sulfide toxicity, in addition to oxidative damage due to the inhibition of antioxidant proteins, may be associated with DNA damage, lipid peroxidation, protein denaturation by disruption of disulfide bonds, and inactivation of redox centers in metalloenzymes [5,12]. At the same time, the protective role of H_2_S against H_2_O_2_ and antibiotics has been demonstrated for different bacteria [6,7,21], which allows us to consider bacterial H_2_S biogenesis as a clinically important adaptive response during infections, capable of acting as a potential therapeutic target [8].

## 3. Sources of Endogenous H_2_S

In mammalian tissues, H_2_S concentrations are maintained at the nanomolar level, while in the intestine they can reach millimolar values (1.0–2.4 mM) [22]. In the intestine, the main source of hydrogen sulfide is commensal sulfate-reducing bacteria, which catalyze the reduction of SO_4_^2−^ to sulfite and then the conversion of SO_3_^2−^ to HS^−^ via dissimilatory sulfite reductase (Dsr) [23]. The most common sulfate-reducing bacteria found in the intestines of humans and animals are the following species: *Desulfotomaculum*, *Desulfobulbus*, *Desulfomicrobium*, *Desulfomonas*, and *Desulfovibrio*, with *Desulfovibrio* (*D. desulfuricans*, *D. farfieldensis*) being the most frequently isolated. These bacteria are not considered directly pathogenic to humans and animals, but the high concentrations of H_2_S they produce may be involved in gut inflammation [22].

In infected mammalian tissues, sources of H_2_S may be the metabolism of host cells and commensal and pathogenic bacteria. Similar to mammalian cells, heterotrophic bacteria endogenously synthesize H_2_S primarily via the enzymes cystathionine β-synthase (CBS) and cystathionine γ-lyase (CSE) or by catabolizing cysteine via cysteine aminotransferase (CAT) to 3-mercaptopyruvate (3-MP), which is then converted to pyruvate and H_2_S by 3-MP sulfotransferase (3MST) via a persulfide intermediate [6,9,10]. Most bacterial genomes contain homologs of the genes encoding CBS/CSE and/or 3MST [6]. For example, *Bacillus anthracis* and *Staphylococcus aureus* have the CBS/CSE operon but lack 3MST [6]. Contrary to what was previously thought, a recent study has revealed that, in addition to CBS and CSE, 3MST also contributes to H_2_S production in *P. aeruginosa* [24]. *S. oneidensis* produces H_2_S through cysteine degradation by methionine γ-lyases MdeA, SO_1095, and SseA [12]. MdeA and SO_1095 are homologs of cystathionine γ-lyase, while SseA is a homolog of 3MST. The *E. coli* genome contains the *mstA* gene encoding 3MST but lacks genes encoding CBS and CSE; however, a mutant lacking 3MST can retain the ability to produce hydrogen sulfide. It was shown that restoration of H_2_S production in this mutant may occur due to a compensatory mutation in the transcriptional repressor *ycjW*, which leads to increased expression of the gene encoding thiosulfate sulfotransferase PspE (rhodanese) [25]. Induction of *pspE* expression in the suppressor strain provides an alternative mechanism for H_2_S biosynthesis.

**Figure 2:**
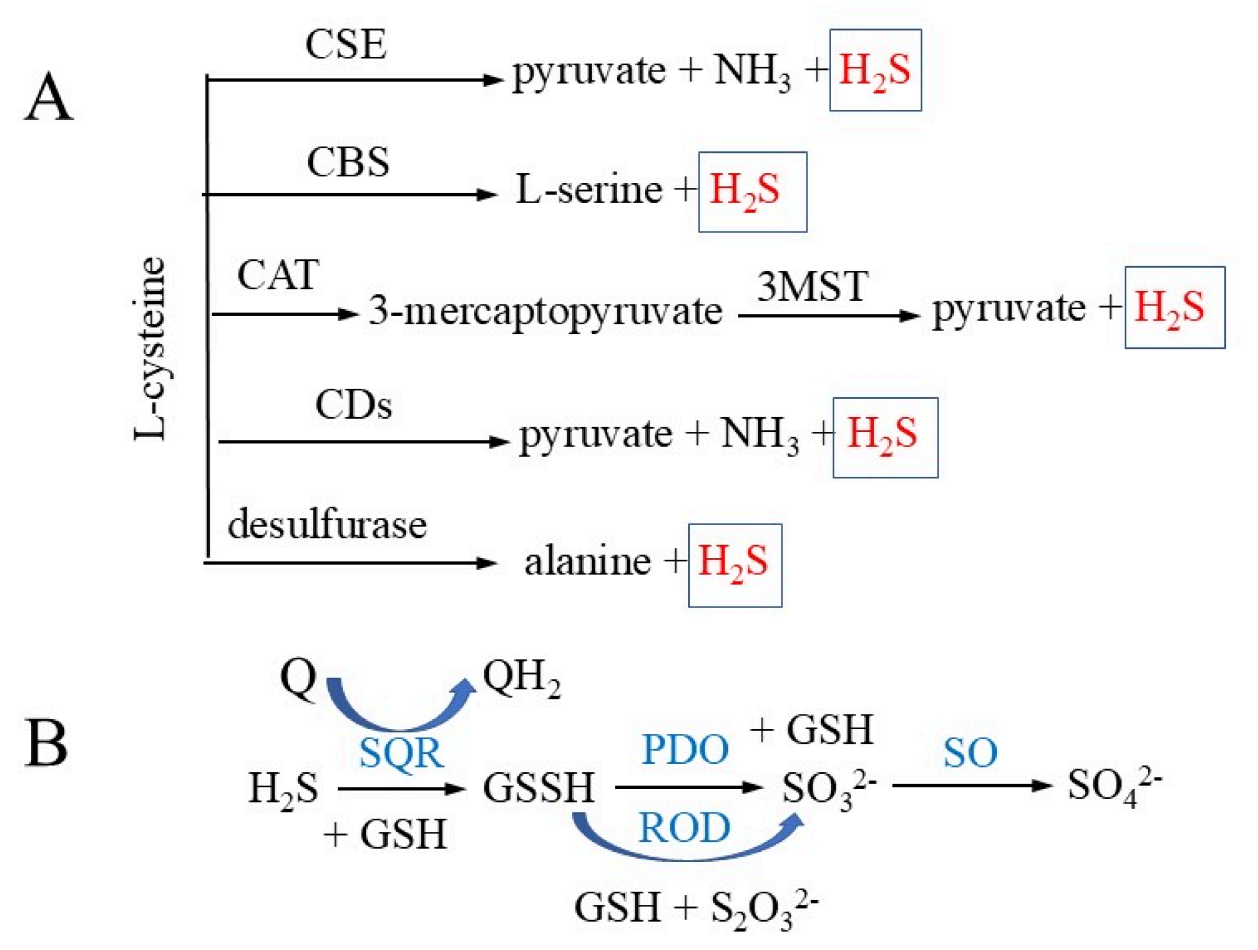
The main pathways of endogenous H_2_S formation (**A**) and modes of its oxidation in heterotrophic bacteria (**B**). CSE—cystathionine γ-lyase; CBS—cystathionine β-synthase; CAT—cysteine aminotransferase; 3MST—3-mercaptopyruvate sulfotransferase; CDs—cysteine desulfhydrases; Q/QH_2_—quinone; GSH—glutathione; GSSH—glutathione persulfide; SQR—sulfide: quinone oxidoreductase; PDO—persulfide dioxygenase; SO—sulfite oxidase; ROD—rhodanese.

In addition to CAT/MST, *E. coli* contains at least six enzymes (CysK, CysM, MetC, TnaA, MalY, and CyuA) with L-cysteine desulfhydrase activity, which can degrade L-cysteine into pyruvate, ammonium, and H_2_S [26,27,28,29]. The functional significance of these enzymes in H_2_S formation remains controversial. However, cysteine desulfhydrases (mainly TnaA) have been shown to be responsible for the degradation of endogenous and exogenous L-cysteine, while 3MST produces sulfane sulfur and is not a major pathway for H_2_S formation [30]. It has also been reported that the main enzyme catabolizing cysteine to produce H_2_S in *E. coli* under anaerobic conditions is cysteine desulfhydrase CyuA, which is controlled by the transcriptional regulator CyuR and is induced by increasing cysteine concentrations [27,28,29]. Furthermore, cysteine desulfurase (IscS), which converts cysteine to alanine and sulfane sulfur, rather than 3MST, was found to be the main source of endogenous H_2_S in *E. coli* under anaerobic conditions [31]. Apparently, H_2_S production in bacteria can be catalyzed by different enzymes depending on the culture conditions (Figure 2A).

The main, although not the only, source of H_2_S in *Mycobacterium tuberculosis* is cysteine desulfhydrase Cds1, which degrades cysteine to produce pyruvate, H_2_S, and ammonium [32]. In *Treponema denticola*, the majority of H_2_S is produced by cystalysins HlyA and HlyB, which are cystathionine β-lyases and hydrolyze cysteine to pyruvate, H_2_S, and ammonium [33]. *Fusobacterium nucleatum* has several enzymes involved in H_2_S production, including L-cysteine desulfhydrase, cysteine synthase, and L-methionine lyase [34]. Furthermore, a glycyl radical enzyme (isethionate sulfite lyase) was discovered from *Bilophila wadsworthia*, which catalyzes the cleavage of the C–S bond during taurine catabolism, producing sulfite, which is then reduced to H_2_S by dissimilatory sulfite reductase [35,36]. Overall, bacteria possess a large arsenal of enzymes capable of producing endogenous sulfide, but the relative contributions and physiological functions of each remain poorly understood.

## 4. Conditions Stimulating Endogenous H_2_S Production

**Figure 3:**
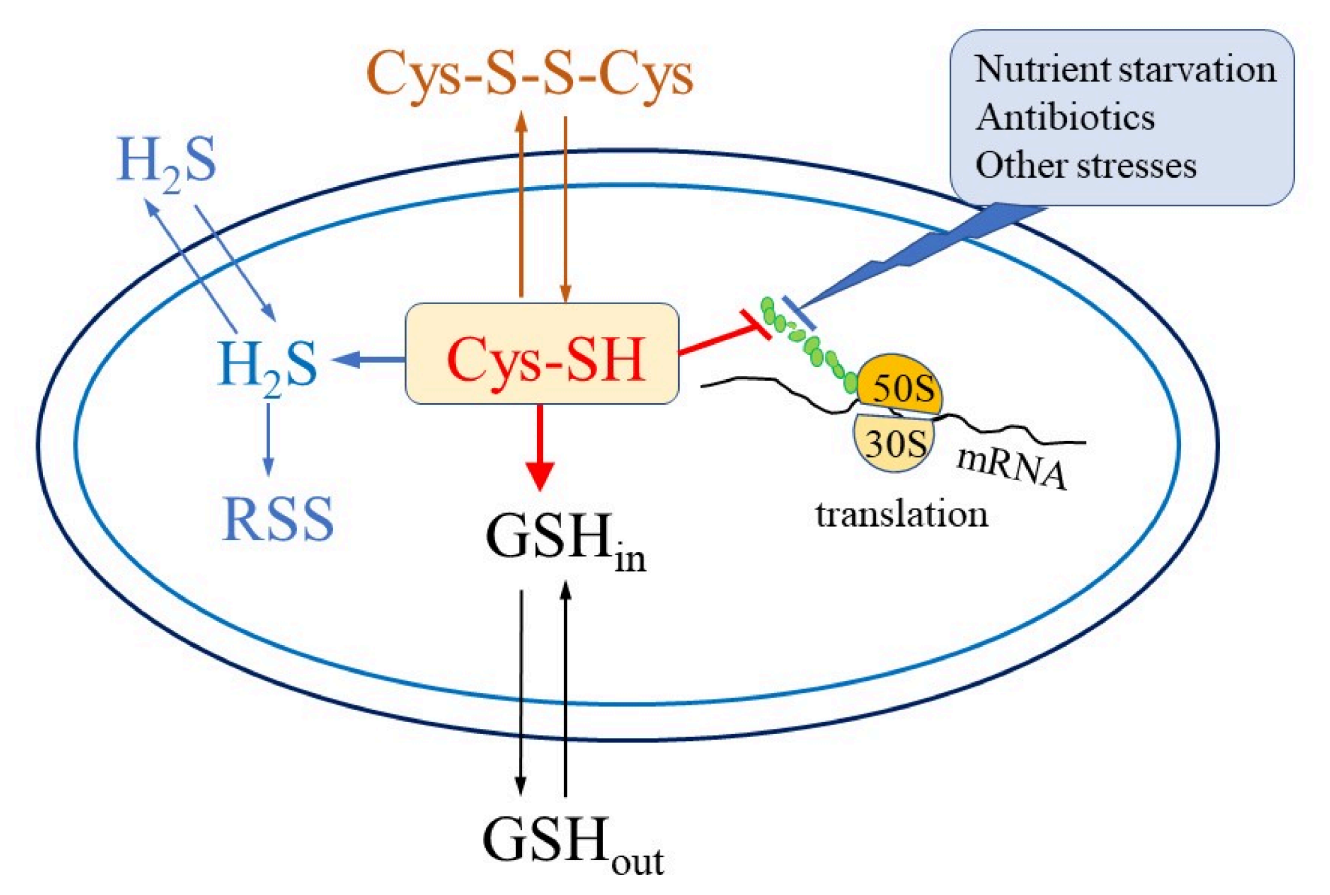
Endogenous H_2_S production by *E. coli* cells in minimal sulfate medium. Stress suppresses protein synthesis. Cysteine, previously involved in protein synthesis, is redirected to glutathione synthesis and is also exported into the medium and partially degraded to form H_2_S. Hydrogen sulfide can freely diffuse across membranes, while GSH and cysteine/cystine undergo transmembrane circulation via their respective transporters. H_2_S oxidation is accompanied by the formation of reactive sulfur species (RSS). Cys-SH—cysteine; Cys-S-S-Cys—cystine; GSH_in_—intracellular glutathione; GSH_out_—extracellular glutathione.

Experiments with continuous monitoring of H_2_S directly in a growing bacterial culture using a sensitive sulfide electrode (sensitivity threshold 5 nM) have shown that endogenous H_2_S production depends on the medium composition, primarily the sulfur source, and cultivation conditions [37]. When grown in a minimal medium, where sulfate is the only sulfur source, *E. coli*, *Bacillus subtilis*, and *Mycobacterium smegmatis* did not release sulfide during exponential growth under aerobic conditions [37,38,39]. Sulfide generation was observed in response to stressful conditions accompanied by a sharp cessation of growth and inhibition of protein synthesis. Such stresses include depletion of glucose, nitrogen, or phosphate in the medium, amino acid starvation, and exposure to a number of antibiotics [18,38,39,40,41,42]. In all these situations, there was a temporary increase in the intracellular concentration of cysteine and activation of its homeostasis mechanisms, including accelerated synthesis of glutathione (GSH) in *E. coli* or mycothiol (MSH) in *M. smegmatis* and the export of cysteine into the medium. Degradation of excess cysteine to form H_2_S can be considered as another mechanism of cysteine homeostasis or as an accidental consequence of an increase in its intracellular concentration (Figure 3).

Activation of these mechanisms protects bacteria from the negative consequences caused by excess cysteine: oxidative stress due to autoxidation reactions, potentiation of the Fenton reaction, and inhibition of isoleucine synthesis [43,44]. The duration and intensity of H_2_S production depended on the bacterial species, the growth rate preceding the stress exposure, and the type of stress. Sulfide production was reduced in the *E. coli mstA* mutant upon exposure to ciprofloxacin and upon ammonium or phosphate depletion, suggesting possible involvement of 3MST in the degradation of excess cysteine in these situations [41,42,45]. The absence of glutathione in the *gshA* mutant stimulated H_2_S generation compared to the parental strain under all stresses studied.

An excess of cytoplasmic cysteine and activation of cysteine homeostasis mechanisms, including H_2_S release, also occur upon addition of cystine to *E. coli* cells growing in minimal sulfate medium [16,46]. With fractional addition of cystine during cultivation, sulfide formation occurred continuously throughout the entire observation period [45]. Deletion of the *cysM*, *mstA*, and *cyuA* genes did not prevent H_2_S formation. It is possible that, under conditions of a strong excess of cytoplasmic cysteine, sulfide formation may be the result of the collective action of all enzymes capable of producing it.

In contrast to sulfate-containing minimal medium, *E. coli* produces H_2_S during normal growth in rich Luria–Bertani (LB Miller) medium (pepton—10 g/L, yeast extract—5 g/L, NaCl—10 g/L), where cystine is the main sulfur source. L-cysteine and L-cystine have been shown to be the most suitable substrates for H_2_S production, while GSH and methionine do not contribute significantly [30]. Simultaneous monitoring of sulfide and O_2_ using electrochemical sensors revealed that the onset of H_2_S production in *E. coli* BW25113 (parental strain) coincides with a sharp, reversible inhibition of respiration at an OD_600_ of approximately 0.35 and is likely associated with metabolic reorganization and growth retardation upon switching to a different substrate in multicomponent LB medium [37,47]. This may indicate a transient process in which excess intracellular cysteine arises, similar to that observed during inhibition of protein synthesis in *E. coli* in minimal medium. Slow cystine import in the *tcyP* and *cysB* mutants, which lack, respectively, the major cystine transport system TcyP and the CysB regulator, which controls both cystine importers TcyP and TcyJLN [45], resulted in almost complete cessation of sulfide production due to the lack of excess cysteine in the cytoplasm of these strains [47]. Mutants *eamA*, *eamB*, *bcr*, and *cydD* lacking cysteine exporters [48,49] exhibited multiple cycles of H_2_S production: the first cycle began immediately after transfer of bacteria to fresh medium, and subsequent cycles were repeated at intervals of 20–30 min, indicating that cysteine export is required for fine-tuning of cytoplasmic cysteine levels [47]. Increased H_2_S production in the *gshA* mutant confirms the important role of glutathione as a cysteine buffer, as it occurs in minimal medium [40]. The highest level of sulfide accumulation in the medium was observed in the *cysK* mutant, which is apparently due to a reduced ability of this strain, lacking the essential cysteine synthase, to incorporate formed H_2_S back into cysteine [47]. H_2_S production in the *mstA* mutant differed little from that in the parental strain. Sulfide production was reduced in the *cysM*, *malY*, *tnaA*, *metC*, *cyuA*, and *iscS* mutants lacking cysteine desulfhydrases, indicating the possible contribution of each of these enzymes to H_2_S formation [47].

Thus, heterotrophic bacteria release sulfide when intracellular cysteine is in excess. When cultivated in a medium where sulfate is the sole sulfur source, disruption of cysteine homeostasis can be caused by the addition of exogenous cystine or various factors that inhibit growth and the synthesis of protein, which is the primary consumer of cysteine synthesized by the cell. In LB, where cystine is a component of the medium, an increase in the intracellular cysteine concentration and, consequently, H_2_S formation can occur without any external factors as a result of metabolic changes and growth retardation. These differences in H_2_S formation, related to medium composition, must be considered when studying the role of endogenous H_2_S in bacterial responses to various factors, including antibiotics.

## 5. Mechanisms of H_2_S Action

In various compounds, sulfur can have oxidation states ranging from −2 to +6. At a sulfur oxidation state of −2, H_2_S and organic thiols (e.g., cysteine or GSH) exist in their most reduced forms and can function solely as cellular reductants [8]. Based on its chemical properties, H_2_S can influence cellular redox physiology through several mechanisms: neutralization of reactive oxygen and nitrogen species, reaction with metal centers of enzymes, modulation of cellular respiration, and post-translational modification of protein SH groups (S-persulfidation) [9].

### 5.1. The Effect of H_2_S on Metalloenzymes

It is well known that H_2_S can interact with metals, including iron, copper, nickel, and zinc, which are components of many enzymes and regulatory proteins through covalent bonding, redox interactions, or coordination [50]. At physiological concentrations, H_2_S can participate in the maintenance of metal homeostasis and signaling through interactions with metalloproteins [51]. Available data indicate that H_2_S may play a key role in attenuating iron cytotoxicity and controlling iron homeostasis under stress. Endogenous H_2_S generation is also often regulated by iron [52].

At the same time, higher concentrations of exogenous H_2_S can disrupt the functions of enzymes containing iron. The effect on cellular physiology may vary depending on the time of H_2_S addition. For example, in the simultaneous presence of H_2_S and H_2_O_2_, an increase in cytotoxicity was observed due to heme damage in catalases, while with sequential addition, H_2_S exerted a protective effect against H_2_O_2_ by activating OxyR and inducing antioxidant defenses [12,23,53]. Proteins modified by H_2_S also include zinc finger (ZF) proteins, which contain cysteine residues that coordinate zinc. In mammals, addition of H_2_S to the ZF protein tristetraprolin under aerobic conditions resulted in rapid persulfidation, thiol oxidation, zinc release, and inhibition of function due to loss of RNA binding capacity [54].

### 5.2. The Effect of H_2_S on Cellular Respiration

Exposure to high concentrations of H_2_S inhibits the mitochondrial cytochrome *aa*_3_ and bacterial heme-copper oxidases but does not affect the *bd*-type oxidases tested so far [55]. The model proposed by Nicholls et al. [56] for the H_2_S-mediated inhibition of the mitochondrial cytochrome *c* oxidase can be extrapolated to bacterial heme-copper oxidases (Figure 1B). According to the model, during steady-state turnover of a heme-copper oxidase, a first molecule of H_2_S (presumably in the form of HS^−^) transiently binds to the Cu_B_ site (whether oxidized or reduced). It then transfers to the ferric iron ion of the high-spin heme (*a*_3_, *o*_3_, or *b*_3_). This transfer blocks the high-spin heme site from reacting with O_2_, halting the enzyme’s function. In this inactive state, the Cu_B_ site is reduced and possibly bound to a second HS^−^ molecule. The inhibition is completely reversible upon removal of H_2_S from the medium [55].

### 5.3. Antioxidant Effects of H_2_S and Persulfides

H_2_S can directly react with reactive oxygen and nitrogen species such as H_2_O_2_, superoxide radical, peroxynitrite, and hypochlorite, acting as an antioxidant [9,57,58]. Oxidation of H_2_S can lead to the formation of a variety of compounds in which the sulfur atom has oxidation states from −2 to +6. Oxidation products include sulfate (SO_4_^2−^), sulfite (SO_3_^2−^), thiosulfate (S_2_O_3_^2−^), persulfides (RSS^−^), organic and inorganic polysulfides, and elemental sulfur. All small inorganic and organic molecules derived from H_2_S and containing sulfur atoms in oxidation states more positive than −2 are collectively referred to as reactive sulfur species (RSS), many of which contain sulfur bound to sulfur, or “sulfane” sulfur. The organic thiol persulfide (hydropersulfide, RSSH) is of particular interest because it can function either as a nucleophile in the deprotonated state (RSS^−^) or as an electrophile in the protonated state (RSSH). Persulfides, formed upon exposure of cells to exogenous H_2_S or during endogenous H_2_S production, readily react with oxidants such as hydrogen peroxide and peroxynitrite and are more effective one-electron reducing agents than thiols and H_2_S [8]. Persulfides are presumed to be responsible for the antioxidant effects observed with H_2_S.

### 5.4. Modification of Protein SH Groups

Persulfidation, the addition of a sulfur atom that converts the thiol group (–SH) of proteins to persulfide (–SSH), is a post-translational modification comparable to nitrosylation or glutathionylation [59]. This modification can protect SH groups from oxidation and alter enzymatic activity, contributing to the regulation of cellular redox homeostasis. H_2_S does not react directly with protein cysteines; the presence of an oxidant (e.g., hydrogen peroxide, peroxynitrite, hypochlorite, lipid peroxides) is required for this reaction to occur. The proposed mechanism for persulfide formation is the reaction of sulfur with oxidized cysteines, such as sulfenic acid (RSOH) or disulfide (RSSR) [57]. Unlike classical cysteine oxidation, the S–S–H bond remains fully reversible and can be regenerated using thioredoxins and glutaredoxins. Overall, evidence is accumulating that persulfidation is a reversible, chemically versatile, and enzymatically regulated redox switch that is at the center of sulfur signaling pathways in mammalian, plant, and bacterial cells [8,23,58,60].

## 6. H_2_S and RSS Homeostasis

To maintain physiological H_2_S levels in a non-toxic range, a balance between its formation and clearance is necessary. H_2_S breakdown occurs via a catabolic pathway in which H_2_S is oxidized to thiosulfate and sulfate in the presence of O_2_. H_2_S oxidation is accomplished by the enzymes sulfide:quinone oxidoreductase (SQR), persulfide dioxygenase (PDO), rhodanese, and sulfite oxidase (SO) [61,62,63]. SQR oxidizes H_2_S to form glutathione persulfide GSSH, and PDO catalyzes the conversion of GSSH to reduced glutathione (GSH) and sulfite (SO_3_^2−^) (Figure 2B). Rhodanese can transfer sulfane sulfur between various acceptors, maintaining a pool of sulfane sulfur in the cytoplasm. It catalyzes the transfer of sulfane sulfur from GSSH to sulfite, resulting in the formation of GSH and thiosulfate, which may help minimize the accumulation of sulfane sulfur in cells and prevent disulfide stress [64]. Conversely, upon addition of exogenous thiosulfate, rhodanese PspE converts it to GSSH in *E. coli* cells, increasing the level of sulfane sulfur, which reduces the sensitivity of bacteria to H_2_O_2_ treatment [65]. Furthermore, sulfane sulfur can be formed during the oxidation of H_2_S involving heme-containing proteins, as well as during cysteine metabolism by various enzymes, including 3MST and desulfurase [58]. Continuous formation and metabolism of sulfane sulfur and H_2_S can maintain their concentrations within a certain range.

The H_2_S/RSS homeostasis mechanism is mediated by RSS sensors, specialized transcriptional regulators that can sense even small changes in cellular RSS caused by endogenous or exogenous perturbations [58]. RSS sensors belong to the copper-sensitive operon repressor (CsoR), arsenic repressor (ArsR), and Fis superfamilies. Most known RSS sensors are characterized by the presence of a pair of cysteines that form a disulfide or polysulfide bridge upon incubation with a sulfane sulfur donor, resulting in the release of repression and transcription of the targeted genes [66]. CstR proteins, which belong to the CsoR superfamily, are found mainly in Gram-positive organisms (Firmicutes) and are best characterized in *S. aureus*, *Streptococcus pneumoniae*, *Streptococcus mitis*, and *Enterococcus faecalis* [51,67]. A typical persulfide sensor belonging to the ArsR family in many Gram-negative organisms is SqrR (sulfide quinone reductase repressor), first characterized in *Rhodobacter capsulatus* [68]. Homologues of SqrR include BigR (biofilm repressor) in *Xylella fastidiosa* and *A. baumannii*, YgaV in *E. coli*, and HlyU in *Vibrio* spp. and probably PigS in *Serratia* [58]. In the chemoorganoheterotrophic bacterium *Hyphomicrobium denitrificans*, the use of thiosulfate as an electron donor is regulated by two sulfane-sulfur-responsive ArsR-type transcriptional repressors, sHdrR and SoxR [69]. The third major class of RSS sensors is represented by the FisR protein from the genus *Cupriavidas*, which is an σ^54^-dependent transcriptional activator [70].

RSS sensors regulate genes whose products are involved in maintaining H_2_S/RSS homeostasis, including SQR, PDO, flavin-dependent coenzyme A persulfide reductase, various sulfotransferases (ST), and one or more membrane transporters, including the putative sulfite exporter TauE and thiosulfate importers YedE/YeeE [58,71]. In addition, so-called secondary RSS sensors, whose primary role differs from H_2_S/RSS homeostasis, are distinguished [58]. They may have a specific input signal distinct from RSS and are involved, for example, in the detection and detoxification of ROS (OxyR, PerR) or in the regulation of virulence genes and can often act as global regulators. The transcriptional regulator OxyR, which responds to increased H_2_O_2_, also senses high RSS levels by activating the expression of thioredoxin, glutaredoxin, and catalase, which can remove sulfane sulfur [53,72]. The PerR protein in the cyanobacterium *Synechococcus* sp., a representative of Cys4 zinc finger proteins, acts not only as a ROS sensor but also as a sensor of RSS, which can modify cysteine in the Cys4:Zn^2+^ structural site to form Cys^121^-SSH, resulting in the release of the zinc atom and activation of the *prxI* gene expression encoding peroxiredoxin [73]. Sulfane sulfur post-translationally modifies the transcription factor AdpA, which controls secondary metabolism and morphological differentiation in *Streptomyces coelicolor*, to form persulfide, thereby stimulating the expression of its target genes, leading to the activation of actinorhodin biosynthesis and morphological changes [74]. In the chemolithotrophic bacterium *Acidithiobacillus caldus*, a novel RSS-sensitive transcription factor of the MarR family (SscR) was identified, which, together with the Cu-sensitive transcriptional repressor CsoR, regulates intracellular RSS levels, allowing *A. caldus* to reduce copper-induced stress and oxidative damage while preventing lethal RSS accumulation [75]. Many of the secondary RSS sensors that play important roles in bacterial virulence and antibiotic resistance are described in the following sections.

In response to exogenous H_2_S, bacteria increase the expression of genes encoding enzymes that oxidize sulfide (PDO, SQR), ensure sulfite export and switch respiration to sulfide-resistant cytochrome *bd*, and inhibit the expression of genes encoding sulfur source transporters [5,17,71]. The number of enzymes involved in ROS detoxification (catalase, superoxide dismutase, alkyl hydroperoxidase, universal stress proteins) and metal metabolism also increases [5,17]. In general, bacterial cells respond to increased H_2_S levels by activating defenses against disulfide and oxidative stress.

When studying the effects of H_2_S and RSS on bacterial virulence and antibiotic sensitivity, researchers use various methods to perturb H_2_S concentrations in cells: decreasing endogenous H_2_S production by deleting or inhibiting enzymes involved in its production from cysteine, or increasing endogenous H_2_S levels by supplementing the medium with cysteine/cystine or deleting enzymes involved in H_2_S degradation, and increasing H_2_S concentrations by adding sources of exogenous H_2_S (NaHS, Na_2_S, gaseous H_2_S, and other chemical donors). Based on the findings described in the preceding sections, different adaptive responses of cells to variations in endogenous and exogenous H_2_S should be expected. Endogenous H_2_S synthesis in heterotrophic bacteria is caused by the accumulation of excess intracellular cysteine, which is also accompanied by the activation of other mechanisms of its homeostasis, such as cysteine export into the medium, incorporation into buffering molecules (GSH, MSH), inhibition of synthesis at the biochemical level, and inhibition of gene expression at the transcriptional level. Suppression of the activity of H_2_S-producing enzymes can, at least in part, be compensated for by the activation of other mechanisms of cysteine homeostasis. Although an increase in intracellular cysteine levels creates a potential risk of oxidative stress, the development of disulfide stress under these conditions is less likely. In contrast, when bacterial cells are treated with sources of exogenous H_2_S, the risk of disulfide stress primarily arises, which triggers systems involved in H_2_S degradation and the restoration of H_2_S/RSS homeostasis. Persulfide formation can occur both during cysteine metabolism and as a result of H_2_S oxidation. However, despite certain parallels, differences in stress responses to increased intracellular cysteine and increased exogenous H_2_S may be the source of differences in the physiological response of bacteria to stress induced by antibiotics and oxidants. Furthermore, the concentration of endogenous H_2_S varies greatly depending on specific conditions, the bacterial species, and their physiological state, whereas the amount of added exogenous H_2_S typically far exceeds the level produced by the cells themselves. Differences in the effects of endogenous and exogenous H_2_S may be one reason for the discrepancies in the results obtained by different researchers.

## 7. The Effect of H_2_S and RSS on Bacterial Virulence

H_2_S and its derivatives, RSS, may promote bacterial survival in the host organism by enhancing resistance to multiple oxidative stressors produced during the antimicrobial immune response and by H_2_S/RSS-dependent regulation of virulence factors and biofilm formation.

### 7.1. Changes in H_2_S Concentration Modulate Bacterial Virulence

The stimulating effect of H_2_S on virulence has been demonstrated in various bacterial species. It has been shown that a decrease in H_2_S production in *E. coli* and *S. aureus* under the influence of specific inhibitors or mutations of H_2_S-producing enzymes increased the susceptibility of both bacterial species to rapid elimination by immune cells, while the addition of exogenous H_2_S mitigated this effect [76]. A *cbs* mutant lacking cystathionine β-synthase, the primary producer of endogenous H_2_S in *Vibrio cholerae*, exhibited a colonization defect in adult mouse experiments, whereas complementation with *cbs* or exogenous addition of N-acetyl-L-cysteine restored its viability in the host environment. According to the model proposed by the authors, the protective effect of endogenous H_2_S may be associated with the detoxification of ROS resulting from the increased post-translational activity of catalase KatB, as well as with the stimulation of the expression of several other antioxidant enzymes (SodB, KatG and AhpC, DNA protection protein DPS and Trx1) [77]. Host-derived H_2_S, like endogenous bacterial H_2_S, stimulated *M. tuberculosis* growth and respiration, primarily through activation of cytochrome *bd* oxidase, and regulated genes involved in sulfur and copper metabolism, as well as the Dos dormancy regulon [32,78,79]. Mice infected with *M. tuberculosis* and deficient in the H_2_S-producing cystathionine β-synthase lived longer, and the use of CBS inhibitors reduced bacterial load, suggesting that methods targeting H_2_S production may be potentially useful for tuberculosis treatment [78].

In *Mycoplasma pneumoniae*, H_2_S, produced by the bifunctional enzyme cysteine desulfurase/desulfhydrase HapE, is a virulence factor that stimulates erythrocyte lysis, inhibits human bronchial epithelial cell proliferation, arrests the cell cycle, and alters the cytokine profile of the local immune system [80]. The spirochete *Treponema denticola* produces H_2_S, which significantly contributes to its pathogenic potential by inducing apoptosis of periodontal epithelial cells and hemolysis of erythrocytes, which may play a key role in the initiation of periodontitis [33].

### 7.2. The Effect of H_2_S/RSS on the Expression of Virulence Factors

The H_2_S signal mediated through RSS can be sensed by global regulators and influence the expression of virulence factors. For example, in *S. aureus*, which is capable of expressing a wide range of virulence genes, it was demonstrated that persulfidation of the transcriptional regulator MgrA inhibits its binding to DNA, leading to altered expression of secreted virulence factors, and modulates secretome cytotoxicity. Exoprotein expression was induced by high sulfide levels and suppressed by low RSS levels [81]. In *V. cholerae*, the transcriptional repressor HlyU (a homolog of SqrR) regulates the expression of exotoxins required for bacterial colonization of the intestine in response to endogenously produced persulfides [82]. The activity of the quorum-sensing regulator LasR was enhanced by sulfane sulfur, which reaches its maximum level in the early stationary phase of *P. aeruginosa* PAO1 [83].

### 7.3. The Effect of H_2_S/RSS on Biofilm Formation

A critical factor for bacterial survival within the host is the formation of biofilms (communities of adherent cells embedded in an extracellular polymer matrix), which provide protection to bacteria from the immune system and antibiotics, making it difficult to eradicate infections [84]. Evidence is accumulating of a link between the regulation of biofilm formation and H_2_S/RSS homeostasis, but this issue remains poorly understood. H_2_S has been detected in the sputum of patients with cystic fibrosis, which represents a complex biofilm [85]. H_2_S has also been found to promote biofilm formation by the intestinal microbiota while simultaneously reducing the proliferation of planktonic cells [86]. Depending on their concentration, endogenous and exogenous H_2_S can differentially affect various characteristics of intestinal mucosal microbiota biofilms, altering the relative abundance, spatial organization, and function of bacteria in biofilms [87]. Low levels of exogenous H_2_S directly stabilize the intestinal mucosa and protect the intestinal microbiota, likely through iron chelation [88]. Mutation of cystathionine γ-lyase and the use of inhibitors that suppress endogenous H_2_S production have also been shown to reduce persister cell formation and biofilm abundance in *S. aureus* and *P. aeruginosa*. Moreover, genes associated with biofilm formation, including genes encoding alginate and other exopolysaccharides, are among the most strongly downregulated categories in H_2_S-deficient cells [89]. The effect of H_2_S on biofilm formation may be mediated through RSS. The secondary persulfide sensor BigR in *A. baumannii* affected the expression of genes associated with biofilm formation; furthermore, the biofilm-regulating transcriptional regulators BfmR and Crp were found to undergo strong persulfidation upon exposure to sulfide [71]. Potential targets of the RSS-sensitive transcription factor SscR in *A. caldus* include diguanylate cyclase and phosphodiesterase, modification of which may influence bacterial biofilm formation by regulating c-di-GMP levels [75].

## 8. The Impact of H_2_S and RSS on Bacterial Sensitivity to Antibiotics

Of particular interest is the relationship between H_2_S production and bacterial sensitivity to antibiotics. Endogenous or exogenous H_2_S has been shown to reduce the sensitivity of *Bacillus anthracis*, *P. aeruginosa*, *S. aureus*, and *E. coli* to antibiotics, while mutations and inhibitors of H_2_S-producing enzymes enhance the lethal activity of antimicrobials [6]. The authors suggested that H_2_S is a universal defense mechanism against antibiotics of different classes (quinolones, aminoglycosides, and β-lactams) in various bacterial species [6]. They also assumed that the main protective mechanism mediated by H_2_S is its participation in maintaining the cellular redox balance, which, according to the radical hypothesis of the action of bactericidal antibiotics, is disrupted due to increased ROS production [90,91,92]. It has been suggested that the protective effect of H_2_S against antibiotics is achieved by stimulating the major antioxidant enzymes catalase and SOD and reducing ROS formation, as well as by reducing cysteine levels and binding free Fe^2+^, which prevents the Fenton reaction that generates toxic hydroxyl radicals [6,7,9,25,93,94]. A model explaining the interaction between L-cysteine metabolism, H_2_S production, and oxidative stress has been proposed, in which 3MST protects *E. coli* from oxidative stress through L-cysteine utilization and H_2_S-mediated scavenging of free iron, which is required for the genotoxic Fenton reaction [7]. However, despite indirect evidence, no study has yet directly demonstrated the involvement of H_2_S in reducing free intracellular iron concentrations [95].

Several studies confirmed the possibility of enhancing the sensitivity of various bacterial species to antibiotics or even restoring antibiotic sensitivity in resistant strains by limiting endogenous H_2_S production. It was shown that chemical inhibition of H_2_S biosynthesis restored antibiotic sensitivity in multidrug-resistant uropathogenic *E. coli* isolates, while exposure to an H_2_S donor was accompanied by restoration of antimicrobial resistance [93]. The authors proposed that the protective effect of H_2_S against antibiotics could be mediated by two mechanisms: (i) down-regulation of energy-efficient cytochrome *bo*_3_ oxidase (CyoA) and induction of less-energy efficient cytochrome *bd* oxidase I/II (CydAB/AppBC) to maintain respiratory flux and redox balance, and (ii) augmentation of antioxidant capacity by elevating catalase and superoxide dismutase activities [93]. The ability of bacteria to maintain respiration and growth in the presence of low micromolar concentrations of sulfide by switching respiration from heme-copper cytochrome *bo*_3_ to cytochromes *bd*-I and *bd*-II [16,17] was discussed above (see also Figure 1). The role of alternative respiratory cytochrome oxidases in H_2_S-mediated bacterial protection from oxidative stress and bactericidal antibiotics was also studied by Seregina et al. [94]. It was shown that deletion of the *cydB* or *cydD* genes leads to hypersensitivity of *E. coli* to quinolones and β-lactams, whereas constitutive expression of the *katG* and *mstA* genes, which ensures increased production of catalase or H_2_S in these mutants, contributed to a decrease in their sensitivity to antibiotics. It has also been noted that H_2_S-mediated activation of catalases and superoxide dismutases may contribute to a decrease in bacterial sensitivity to antibiotics [96,97].

The effect of H_2_S on bacterial antimicrobial resistance may be mediated by RSS signaling, including the regulation of resistance gene expression. Growth-related changes in sulfane sulfur content have been shown to regulate the expression of antibiotic resistance genes controlled by transcriptional regulators of the MarR (multiple antibiotic resistance regulators) family [98]. These regulators are ubiquitous in bacteria, modulating numerous cellular processes, and are well known for their ability to induce resistance to multiple antibiotics, detergents, and oxidative reagents. Although several inducers have been reported, sulfane sulfur, whose concentration peaks in *E. coli* cells during the late logarithmic and early stationary phases of growth, is likely a key inducer regulating MarR activity. Persulfidation of thiols within the MarR protein alters its structure and affinity for DNA, leading to the derepression of the genes it controls [98]. Persulfides have also been shown to directly react with SH groups of the MexR transcriptional regulator when *P. aeruginosa* PAO1 enters stationary phase, leading to the derepression of the *mexAB-oprM* operon and the activation of the MexAB-OprM efflux pump, which plays a critical role in antibiotic resistance [99]. The MgrA regulator is modified by sulfane sulfur when *S. aureus* is exposed to H_2_S-induced stress, derepressing genes involved bacterial virulence [81]. Both MexR and MgrA belong to the MarR family. Thus, three family members sense the presence of sulfane sulfur in cells, activating antibiotic resistance genes, further confirming the signaling role of sulfane sulfur in bacteria.

In *E. coli*, the sulfide-responsive transcription factor YgaV (a homolog of SqrR/BigR) controls the expression of anaerobic respiration genes and is involved in H_2_O_2_ scavenging [100]. The *ygaV* mutant was more sensitive to antibiotics than the parental strain and, unlike it, did not respond to the presence of H_2_S with decreased sensitivity, indicating an important role for YgaV-dependent transcriptional regulation in maintaining redox homeostasis, ROS scavenging, and sensitivity to antibiotics [100].

Furthermore, it has been found that persulfides can participate in the direct degradation of some antibiotics. In particular, cysteine hydropersulfide (CysSSH), formed by the reaction of H_2_S with cystine, can directly inactivate β-lactam antibiotics of the penicillin (penicillin G and ampicillin) and carbapenem (meropenem) classes, forming ring-opened β-lactam S-acids (BL-COSH) [101]. Since *E. coli* and *S. aureus* efficiently degraded β-lactam antibiotics to form BL-COSH, the authors suggested that CysSSH-mediated degradation of β-lactams may be one of the mechanisms reducing their sensitivity to these antibiotics.

Most of the mechanisms considered for the protective effect of H_2_S against antibiotics are still hypothetical and require further in-depth research across species and systems. The hypothesis of oxidative stress as a general mechanism of action of bactericidal antibiotics has been challenged [102]. Accordingly, the involvement of H_2_S and its derivatives as antioxidants against antibiotic-generated ROS is also questionable. However, antibiotics can disrupt cysteine homeostasis as part of a universal stress response associated with the inhibition of protein synthesis [45,47]. H_2_S and its derivatives may be involved in maintaining cysteine homeostasis and cellular redox homeostasis in general. The involvement of H_2_S and RSS in cellular signaling and the activation of antibiotic resistance gene expression through persulfidation of transcriptional regulators is a very attractive mechanism but also requires further research. H_2_S-induced respiration switching from cytochrome oxidase *bo*_3_ to *bd* requires the production of micromolar concentrations of endogenous H_2_S, which may depend on the bacterial species and their physiological state.

The potential for using H_2_S production inhibitors as adjuvants for widely used antibiotics has generated considerable interest among researchers, who have sought to identify such compounds. Several compounds have been identified that enhance the antimicrobial activity of known antibiotics (Table 1). In particular, pioglitazone, a specific inhibitor of the 3MST enzyme in *E. coli*, suppressed H_2_S and sulfane sulfur production and enhanced the antimicrobial activity of gentamicin and macrophages against this bacterium [103]. Small molecules **NL1**, **NL2**, **NL3** that inhibit cystathionine γ-lyase, the primary H_2_S generator in *S. aureus* and *P. aeruginosa*, have been discovered [89]. These inhibitors enhanced the bactericidal activity of antibiotics against these pathogens both in vitro and in mouse infection models, disrupting biofilm formation and reducing tolerance to antibiotics and the number of persister cells. It was also found that inhibition of cystathionine γ-lyase using naphthyl-substituted indole and pyrrole carboxylic acids significantly enhances the effect of antibiotics against *A. baumannii*, *P. aeruginosa*, and methicillin-resistant *S. aureus* [104]. Furthermore, compound **7b**, based on nitrobenzofuran structures and capable of scavenging H_2_S, reducing its concentration, enhanced the activity of macrophages and neutrophils, suppressed biofilm formation, and also significantly increased the antimicrobial efficacy of gentamicin in models of pneumonia and skin wounds caused by *P. aeruginosa* [105].

However, despite intensive research into the role of H_2_S and the mechanisms by which it influences bacterial sensitivity to antibiotics, consensus remains elusive. Several research groups have demonstrated that the protective effect of H_2_S is not universal across all bacterial species and antibiotics. Analysis of the effect of H_2_S on the activity of antibiotics of key classes against *S. aureus* revealed that exogenous H_2_S exerts a protective effect only against aminoglycosides, whose transport into the cell depends on ΔμH^+^. The effect of sulfide on the bacterial respiratory chain leads to decreased uptake of aminoglycosides, weakening their antimicrobial effect [106]. It has also been shown that a deficiency in H_2_S production in the same strain can increase sensitivity to one type of antibiotic but does not affect sensitivity to other antibiotics. In particular, the *megL* mutant of *F. nucleatum*, lacking the essential H_2_S-generating enzyme L-methionine γ-lyase, exhibited increased sensitivity to nalidixic acid but demonstrated resistance to kanamycin [107]. Furthermore, the effect of H_2_S on antibiotic sensitivity may be highly dependent on the metabolic characteristics of the bacterial species under study. Instead of decreasing antibiotic sensitivity, exogenous H_2_S increases the sensitivity of *A. baumannii* to several classes of antibiotics and can reverse acquired resistance to gentamicin [11]. This hypersensitivity was presumably caused by H_2_S-induced disruption of energy metabolism and redox homeostasis in this bacterium. In *M. tuberculosis*, endogenous and exogenous H_2_S stimulates respiration and growth, regulates redox homeostasis, and increases sensitivity to the anti-tuberculosis drugs clofazimine and rifampin [32]. A recent study using mutants producing high or low levels of H_2_S showed that H_2_S production is not a protective mechanism against antibiotics of different classes in *P. aeruginosa* [24], which contradicts previously published data [6]. Moreover, a correlation analysis of a large collection of *P. aeruginosa* isolates from patients with cystic fibrosis did not reveal a protective role of H_2_S against antibiotic action during chronic pulmonary infection [24].

It was shown that *E. coli* mutants with defects in cysteine synthesis and degradation (*cysK*, *cysM*, *cyuA*, *iscS*, *malY*, *metC*, *mstA*, *tnaA*), cystine import and cysteine export (*tcyP*, *eamA*, *eamB*, *bcr*, *cydD*), glutathione synthesis (*gshA*), and regulatory proteins (*cyuR* and *cysB*) generated different amounts of H_2_S when grown in LB medium, where cystine is the main source of sulfur [47]. In addition to differences in H_2_S production, mutations caused changes in a range of other parameters: growth and respiration rates, glutathione and cysteine levels, and the degree of induction of the Fur, OxyR, and SOS regulons. A correlation was found between the expression of the Fur-controlled *iucC*::*lacZ* fusion and H_2_S production (r = −0.71, *p* < 0.05), indicating coordinated regulation of the free Fe^2+^ and H_2_S pools. Over a wide range of fluoroquinolone ciprofloxacin concentrations (0.03–10 µg/mL), a significant positive relationship was observed between the level of endogenous sulfide and the specific growth rate of bacteria (μ) after the addition of the antibiotic. However, no statistically significant relationship was found between the rate of bacterial killing in the CFU assay and H_2_S production. At a ciprofloxacin dose of 0.3 µg/mL, a strong direct correlation was found between the rate of bacterial death during the rapid killing phase (30 min) and the intracellular and extracellular glutathione concentrations (r = 0.8, *p* < 0.05). This effect was absent at higher antibiotic concentrations. This may indicate that changes in the redox status of low-molecular-weight thiols during ciprofloxacin-induced perturbations of cysteine homeostasis may contribute to the increased lethal activity of low doses of the antibiotic. At all ciprofloxacin concentrations studied, a strong inverse relationship was observed between the logarithm of CFU/mL of the studied mutants and the specific bacterial growth rate before the addition of the antibiotic. Apparently, mutants that retained higher metabolic activity when exposed to ciprofloxacin suffered more DNA damage, which complicated its repair upon resumption of growth on agar plates.

Treatment of *E. coli* growing in minimal medium with sulfate as the sole sulfur source with ciprofloxacin resulted in the release of H_2_S in the parental strain. The *cysM* and *mstA* mutants did not produce H_2_S, while the *gshA* mutant produced it in higher quantities than the parent. However, in the CFU assay, the *mstA* mutant was less sensitive, while the *cysM* and *gshA* mutants were more sensitive to ciprofloxacin than the parental strain, which is inconsistent with the hypothesis that H_2_S plays a protective role against the bactericidal action of quinolones [45]. At the same time, under conditions of continuous H_2_S release induced by the fractional addition of cystine, all the studied strains were lower sensitive to ciprofloxacin than in a medium without cystine, with the exception of the *gshA* mutant, the sensitivity of which increased 5–8 times at high doses of the antibiotic, indicating the important role of GSH in restoring the redox balance in cells growing in the presence of cystine.

To better understand the possible reasons for the existing contradictions, we compiled a summary table systematizing the available studies by bacterial species, H_2_S perturbation modes, antibiotic classes, experimental conditions, and observed phenotypes (Table 2). To facilitate understanding of the phenotypic effect of antibiotics, we used the unified term “change in antibiotic sensitivity”; however, since the authors employed different experimental approaches, the original papers may have referred to changes in antibiotic sensitivity, tolerance, persistence, or resistance. The analysis showed that certain contradictions exist for most of the studied species. They may be associated not only with the characteristics of the strains and the mechanism of action of antibiotics, but also with differences in the test systems used, as well as with specific cultivation conditions, sources and concentrations of H_2_S and antibiotics, and the physiological state of the bacteria. Some observations appear curious. A specific bCSE inhibitor (compound **2a**) has been shown to increase the sensitivity of *A. baumannii* to β-lactams (Mer, CP, Pip + Taz) [104], while other studies claim that this bacterium maintains very low levels of H_2_S and, although it has 3MST, does not contain bCSE homologues in its genome [11,71].

## 9. Concluding Remarks

**Figure 4:**
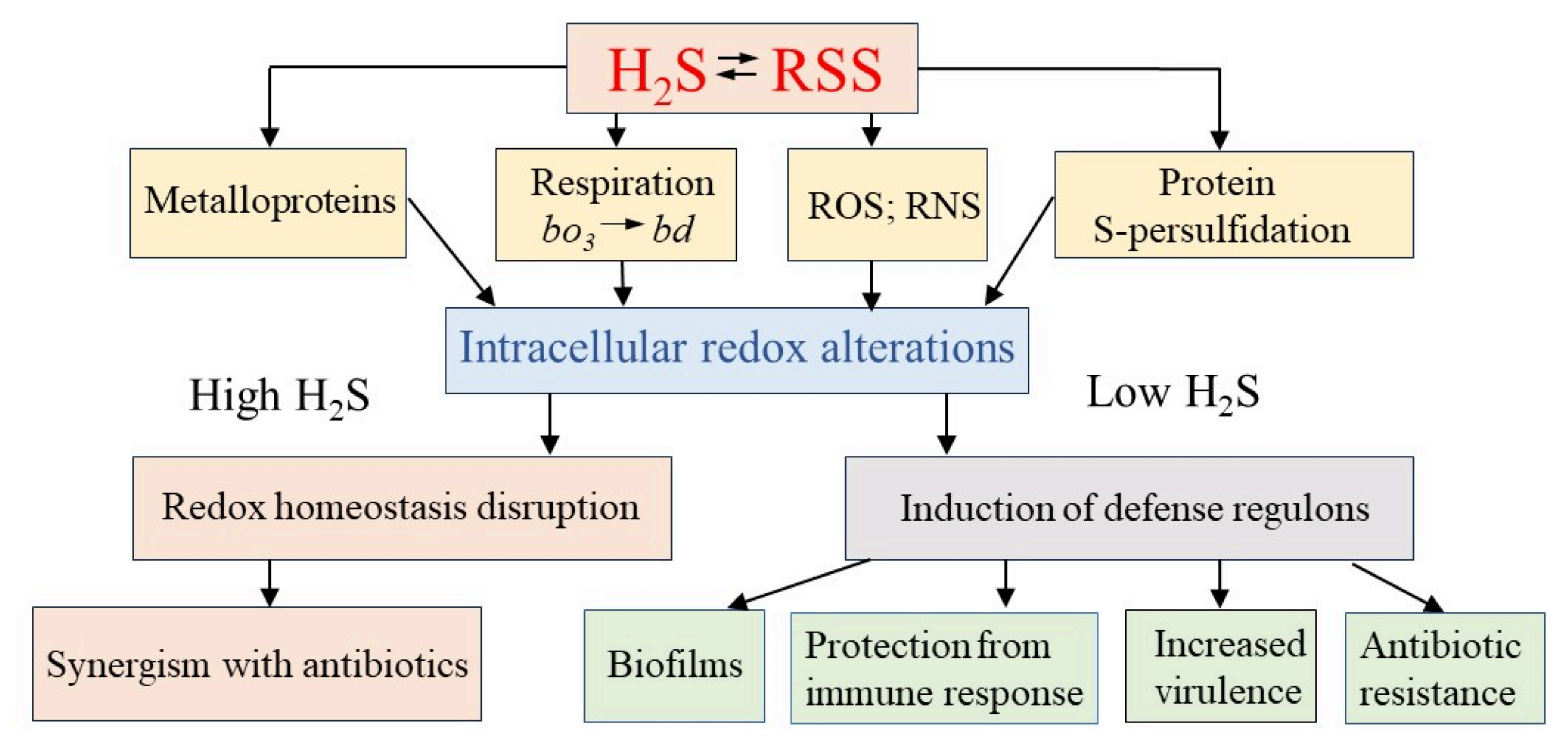
Proposed mechanisms of influence of H_2_S and reactive sulfur species (RSS) on bacterial virulence and antibiotic sensitivity. ROS—reactive oxygen species; RNS—reactive nitrogen species; *bo*_3_—heme-copper cytochrome oxidase; *bd*—copper-lacking cytochrome oxidases.

Literature analysis shows that low doses of exogenous H_2_S may induce regulatory redox changes in transcription factors and enzymes, accompanied by activation of bacterial defense systems and decreased sensitivity to oxidants and antibiotics. High doses of H_2_S, however, irreversibly disrupt the redox balance, promoting cell death, and can act synergistically with antibiotics (Figure 4). The dose required to induce a given effect can vary depending on the bacterial species and physiological state.

The production of endogenous H_2_S in heterotrophic bacteria is in most cases a consequence of increased intracellular cysteine concentrations under conditions that inhibit growth and protein synthesis, including various stresses and antibiotic exposure. Concurrently with H_2_S, the concentrations of key redox buffers (GSH and MSH) and, likely, reactive sulfur species increase, which may lead to changes in the expression of virulence and antibiotic resistance genes. A number of studies in vitro systems and mouse models have demonstrated the possibility of influencing virulence and antibiotic sensitivity in bacteria of various species, including multidrug-resistant strains, by targeted modulation of H_2_S levels. However, limitations related to the lack of standardization of donor systems for H_2_S, differences between culture media and assay systems, uncertainty in physiological sulfide concentrations, and the challenge in separating direct effects from RSS-mediated reactions lead to inconsistent results among different researchers. Many questions remain regarding the mechanism of H_2_S action, the influence of concentrations, culture conditions, specific stress responses in different bacterial species, interactions with host H_2_S, and other issues that need to be addressed, as they may require certain precautions and limitations when manipulating thiol levels during antibiotic therapy. The presence of alternative pathways for the production of endogenous H_2_S and RSS and the multiple mechanisms regulating cysteine homeostasis in cells of the same bacterial species, as well as differences in regulatory mechanisms across species, may require an individualized approach taking into account the metabolic characteristics of specific pathogens. Addressing these issues and elucidating the mechanisms by which H_2_S and RSS modulate the expression of bacterial resistance genes may facilitate the development of new therapeutic approaches based on the regulation of H_2_S and low-molecular-weight thiols, which is particularly relevant in the context of the widespread prevalence of antibiotic-resistant pathogens. However, addressing these issues presents significant challenges due to the strong context dependence of H_2_S/RSS effects, which are largely determined by bacterial physiology and experimental conditions.

## Figures and Tables

**Table 1:** Potential adjuvants that enhance antibiotic activity by reducing H_2_S levels.

Compound	Mechanism of Action	Antibiotics Tested	References
Pioglitazone	specific thiazolidinedione-type inhibitor of *E. coli* 3MST	gentamycin	[103]
Indole-containing compounds **NL1, NL2, NL3**	selective inhibitors of bacterial cystathionine γ-lyase (bCSE) in *S. aureus* and *P. aeruginosa*	ciprofloxacin norfloxacin gentamycin kanamycin ampicillin	[89]
Naphthyl-substituted indole and pyrrole carboxylic acids Lead compound **2a**	selective inhibitors of bCSE in *S. aureus* ATCC 25923 *S. aureus* MRSA *P. aeruginosa* *A. baumannii*	kanamycin norfloxacin ampicillin cefepime meropenem piperacillin + tazobactam	[104]
Compound **7b**, based on nitrobenzofuran structures	H_2_S scavenger in *P. aeruginosa*, *S. aureus*, *S. aureus* MRSA and *E. coli*	gentamycin ciprofloxacin ampicillin vancomycin erythromycin	[105]

**Table 2:** Summary of the effects of H_2_S level perturbations on bacterial sensitivity to antibiotics.

Bacterial Species/Strains	Modes of H_2_S Perturbation	Antibiotics Used	Experimental Conditions and Tests	Observed Phenotype	References
*E. coli* MG1655 *E. coli* MG1655 Uropatogenic *E. coli* (MDR) *E. coli* BL21(DE3), O111:B4 *E. coli* MG1655 *E. coli* BW25113 *E. coli* *ATCC25922* *E. coli* BW25113 *E. coli* BW25113	Reducing H_2_S levels by *mstA* deletion	quinolones aminoglycosides macrolides β-lactams polymyxins antifolates rifamycin	phenotype MicroArray	growth suppression compared to wt	[6]
		NA, Gm, Amp	time-kill assay (LB)	increased antibiotic and H_2_O_2_ sensitivity	[6]
	Restoration of H_2_S levels due to a suppressor mutation in the Δ*mstA* strain	NA, Gm, H_2_O_2_	time-kill assay (LB)	decreased antibiotic and H_2_O_2_ sensitivity compared to Δ*mstA*	[25]
	Reducing H_2_S levels using 3MST inhibitors	NA, Gm, Amp	time-kill assay (LB + aspartate)	increased antibiotic sensitivity	[6]
		Amp	growth (OD_600_) (LB + aspartate)	growth inhibition of multidrug-resistant isolate	[93]
		Gm	time-kill assay (LB + pioglitazone)	increased sensitivity to antibiotics and macrophages; enhanced ROS	[103]
	Increasing H_2_S levels by adding exogenous H_2_S donors	NA, Gm, Amp	time-kill assay (LB + 0.2 mM NaHS)	decreased antibiotic sensitivity	[6]
		Amp, Amik, CF, H_2_O_2_	time-kill assay (LB + NTR-activated H_2_S donor **1c**)	decreased antibiotic and H_2_O_2_ sensitivity; switching from *bo_3_* to *bd*-type cytochrome;	[93]
		Amp, Km, Sm, Tet	antibiotic-saturated paper disks (LB plates + gaseous H_2_S)	decreased sensitivity to antibiotics, except Km	[100]
		Km, Sm	CFU assay 30 min after adding antibiotic (LB + 0.2 mM Na_2_S)	decreased antibiotic sensitivity	[100]
	Reducing H_2_S levels with H_2_S scavenger 7b	Gm, CF	time-kill assay (MHB)	increased sensitivity to antibiotics and macrophages	[105]
	Alteration of H_2_S levels over a wide range in mutants *cysK*, *cysM*, *cyuA*, *iscS*, *malY*, *metC*, *mstA*, *tnaA*, *tcyP*, *eamA*, *eamB*, *bcr*, *cydD*, *gshA*, *cyuR*, *cysB*	CF	MIC, specific growth rate (μ) and time-kill assays (LB)	positive correlation between sulfide levels and μ after antibiotic addition; no significant correlation between bacterial killing rate and H_2_S production; strong inverse correlation between logCFU/mL and μ before CF addition	[47]
	Increasing H_2_S production due to fractional addition of cystine	CF	specific growth rate (μ) and time-kill assays (minimal M9 medium + Cys)	decreased antibiotic sensitivity in all strains studied, except Δ*gshA*	[45]
*P. aeruginosa* PA14 *P. aeruginosa* PAO1 *P. aeruginosa* PAO1 *P. aeruginosa* *P. aeruginosa* PA14 and PAO1 *P. aeruginosa* clinical isolate *P. aeruginosa* PAO1	Reducing H_2_S levels by *csb*/*cse* deletion	NA, Gm, Amp, Nor	MIC and time-kill assays (LB)	increased sensitivity to H_2_O_2_ and antibiotics	[6,89]
	Reducing H_2_S levels by *csb*, *cse*, *mst*, and *cysI* deletion	Tet, Mox, Cef, Car, Cam	antibiotic-saturated paper disks (LB agar plates)	increased antibiotic sensitivity and repression of *mexAB-oprM* multidrug efflux operon, which are reversible by H_2_S	[99]
	Reducing (Δ*3mst cbs cse*) or increasing (Δ*sqr1 sqr2 pdo*) H_2_S production in triple mutants	Car, Cam, CF, Gm, Nor, Tet, Col, Mer, Tob	MIC assays (MHB, or TSB-cys) growth (OD_600_) (LB or LB + NaHS)	MICs of all antibiotics for the mutants were the same as for PAO1. Comparable growth curves of parental and mutant strains treated with sub-MIC concentrations of all antibiotics	[24]
	Clinical isolates with different H_2_S and antibiotic resistance levels		LB	Analysis of 100 clinical isolates revealed that H_2_S levels are lower in resistant and MDR isolates relative to sensitive ones.	[24]
	Reducing H_2_S levels using CSB/CSE inhibitors	NA, Gm, Amp	time-kill assay (LB + PAG/AOAA)	increased antibiotic sensitivity	[6]
		CF, Nor, Amp, Gm. Kan, Tet, Cam	MIC and MBC assays, growth (OD_600_), time-kill assay, lung infection model (LB + NL1, NL2 or NL3)	increased sensitivity to antibiotics, except Tet and Cam; reduced formation of persister cells and biofilms; increased efficacy of Gm + NL1 in a lung infection model	[89]
		Mer, CP, Pip + Taz	MIC assay (LB + compound **2a**)	increased antibiotic sensitivity	[104]
	Reducing H_2_S levels with H_2_S scavenger 7b	Gm, CF	time-kill assay mouse models (MHB + compound **7b**)	increased sensitivity to antibiotics and macrophages; biofilm destruction; increased efficacy of Gm + 7b in mouse model	[105]
*S. aureus* RN4220, MRSA MW2, USA300, Newman *S. aureus* ATCC 25923, INA00761 (MRSA) *S. aureus* ATCC 29213 *S. aureus* HG003, RN4220, Newman, USA300	Reducing H_2_S levels by csb/cse deletion	NA, Gm, Amp, Nor	MIC and time-kill assays (LB)	increased antibiotic and H_2_O_2_ sensitivity	[6,89]
	Reducing H_2_S levels using CSB/CSE inhibitors	NA, Gm, Amp	time-kill assay (LB + PAG/AOAA)	increased antibiotic sensitivity	[6]
		CF, Nor, Amp, Gm. Kan, Tet, Cam	MIC and MBC assays, growth (OD_600_), time-kill assay, murine sepsis model (LB + NL1, NL2 or NL3)	increased sensitivity to antibiotics, except Tet and Cam; reduced formation of persister cells and biofilms; increased efficacy of Gm + NL1 in a sepsis model	[89]
		Kan, Amp, Nor	MIC assay (LB + compound **2a**)	increased antibiotic sensitivity	[104]
	Reducing H_2_S levels with H_2_S scavenger 7b	Gm, CF, Amp, Van, Ery	time-kill assay (MHB + compound **7b**)	increased sensitivity to antibiotics, photodynamic therapy and macrophages; biofilm destruction	[105]
	Increasing H_2_S levels by adding exogenous H_2_S donors	Fox, Sam, Lvx, Tob, Gm, Van, Tet, Tec, Dap, Tgc, Dox, QD, Lin, Ery, Tmp, Sxt, Cam	disk diffusion assay (MH agar plates + H_2_S gaseous); checkerboard microdilution assays with Gm, Amp and AOAA (LB); time-kill assay (LB + Na_2_S)	Sulfide protection was limited to aminoglycoside antibiotics. In the checkerboard analysis, no synergistic effect was found between Gm, Amp and AOAA. Low level of endogenous H_2_S production.	[106]
*B. anthracis* Sterne	Reducing H_2_S levels by csb/cse deletion or with inhibitors PAG/AOAA	NA, Gm, Amp, Nor	MIC and time-kill assays (LB)	increased sensitivity to H_2_O_2_ and antibiotics, reversible with NaHS	[6]
*A. baumannii* ATCC BAA-2093^TM^, clinical isolate #8879 *A. baumannii* ATCC 17978 *A. baumannii* GIMC5509:ABT-52Ts19	Increasing H_2_S levels by adding NaHS	Gm, Col, Rif, Clar	time-kill assay (LB + 80 or 160 μM NaHS)	Absence of *csb*, *cse*, *mst* homologs; absence of endogenous H_2_S. Exogenous H_2_S sensitized *A. baumannii* to multiple antibiotic classes, triggers a prooxidant redox disbalance, reduces membrane potential and ATP level.	[11]
	Deletion of 3MST or addition of Na_2_S (0.2 mM)		LB	Δ*MST* does not directly affect H_2_S levels, but it does reduce other forms of endogenous sulfur. Addition of Na_2_S leads to an increase in LMW thiol persulfide (GSSH) pools and protein persulfidation, including the biofilm response regulator, BfmR. Na_2_S induces CydAB expression and increases the levels of enzymes involved in sulfur and metal homeostasis and ROS detoxification.	[71]
	Reducing H_2_S levels using bCSE inhibitor compound **2a**	Mer, CP, Pip + Taz	MIC assay (LB + 2a)	increased antibiotic sensitivity	[104]
*F. nucleatum* ATCC 23726	Reducing H_2_S levels by *megL* deletion	Amp, Cam, Kan, NA, Met	growth (OD_600_), anaerobic conditions (TSPC)	increased sensitivity to NA, but decreased sensitivity to Kan; attenuated virulence in a mouse model of preterm birth	[107]
*M. tuberculosis* CDC1551, TKK-01-0027, TKK-01-0047, TKK-01-0035, TKK-01-0001	Reducing H_2_S levels by *cds1* deletion	Cfz	CFU-based survival (Middlebrook 7H9 media)	decreased sensitivity of Δ*cds1* to Cfz versus wt. Endogenous H_2_S stimulates respiration via cytochrome *bd* and exacerbates oxidative stress in the presence of CFZ.	[32]
	Increasing H_2_S levels by adding NaHS	Cfz, Rif, INH	CFU-based survival (Middlebrook 7H9 media)	increased sensitivity to Cfz and Rif, but not to INH.	[32]

Antibiotics and their targets: NA—nalidixic acid, CF—ciprofloxacin, Nor—norfloxacin, Lvx—levofloxacin (quinolones, DNA synthesis); Rif—rifampicin (RNA synthesis); Gm—gentamicin, Amik—amikacin, Km—kanamycin, Sm—streptomycin, Tob—tobramycin (aminoglycosides, protein synthesis); Tet—tetracycline, Tgc—tigecycline, Dox—doxycycline (tetracyclines, protein synthesis); QD—quinupristin-dalfopristin (streptogramins AB, protein synthesis); Lin—lincomycin (lincosamides, protein synthesis); Cam—chloramphenicol (protein synthesis); Ery—erythromycin, Clar—clarithromycin (macrolides, protein synthesis); Amp—ampicillin, Mox—moxalactam, Cef—cefsulodine, Car—carbenicillin, Mer—meropenem, CP—cefepime, Pip + Taz—piperacillin + tazobactam, Fox—cefoxitin, Sam—ampicillin-sulbactam (β-lactams, cell wall synthesis); Col—colistin (polymyxins, membrane destruction); Van—vancomycin, Tec—teicoplanin, Dap—daptomycin (glycopeptides, cell wall synthesis); INH—isoniazid (cell wall synthesis); Tmp—trimethoprim, Sxt—trimethoprim-sulfamethoxazole (folic acid inhibitors); Cfz—clofazimine (inhibition of replication and transcription, ROS production). LB—Luria–Bertani medium, MHB—Mueller-Hinton broth, TSB-cys—tryptic soy broth supplemented with L-cysteine, TSPC—tryptic soy broth supplemented with 1% Bacto peptone plus 0.25% freshly made cysteine; PAG—DL-propargylglycine; AOAA—aminooxyacetate.

## Data Availability

No new data were created in this study. Data sharing is not applicable to this article.
